# Congenital Non-Expansion of the Lungs

**Published:** 1856-12

**Authors:** G. A. Kunkler

**Affiliations:** Madison, Ia.


### ON THE CONGENITAL NON-EXPANSION OF THE LUNGS. (ATELECTASIS.)

BY G. A. KUNKLER, M. D., OF MADISON, IA.

During January, 1855, I attended Mrs. J. T—— in her second confinement. The labor was rapid, and completed without any untoward circumstance. The child was large and well developed in every respect. It was noticed, however, that immediately after its birth, its movements were very feeble; the voice was hoarse and weak, more resembling a whine. The breath was very short, the child occasionally making convulsive gasps, and the thorax scarcely moving during the inspirations. The skin had a bluish cast. After being put to the breast, it would cease every few moments from suckling, to breathe. It was placed in a warm bath, and immediately some improvement took place; the skin became of a more natural color; the movements of the limbs less feeble; the voice louder, but the respiration remained as before. On taking it out of the bath, all the previous symptoms re-appeared. On percussion, and applying the ear, the lungs appeared to be acting only in small circumscribed regions; the signs resembling those that are observed in pneumonia after hepatization has taken place.

The symptoms during the day gradually became more aggravated; convulsions set in; the skin becoming bluish-purple and clammy, and the voice hoarse and croupy. These paroxysms lasted from two to five minutes, and generally returned whenever the child commenced to suckle, or was moved about a great deal.

Presuming that the symptoms were caused by a greater portion of the lungs having remained in the fœtal state, I adopted the treatment which I had found most effectual in two other cases which came under my observation, and which was most likely to make the cells of the lung accessible to the ingress of air. The child was placed in a warm bath, and friction freely applied over the whole surface. The thorax was next lifted above the surface of the water and sulphuric ether freely sprinkled over it and allowed to evaporate. The effect was rapid; the child made violent efforts to take deep inspirations, and commenced to scream with all its power. The voice became gradually more full and strong; the thorax expanding with every inspiration; the color of the skin becoming natural, and the movements of the limbs more energetic. Upon examining the lungs afterwards, the respiratory murmur was heard in every part. No further symptoms showed themselves, and the child has ever since enjoyed good health.

*Remarks.*—The above case I have considered a fair example of that peculiar lesion which, at various times, has been the cause of extensive controversies, some contending that the lesion is the consequence of pneumonic inflammation; others, that the deficient expansion is caused by an enlargement ot the thymus gland, etc. The most complete, accurate, and learned treatise, one which, in my opinion, places the true nature of the lesion beyond all doubt, was published in 1832, by Edward Jorg,* a German physician, who, following out some views of his father which had been published, more closely investigated that particular condition of the lungs of new-born children found after death, in cases where the first act of breathing has been imperfectly accomplished, either because they were feeble, or had been born before placental respiration was altogether suspended, and before the necessity for pulmonary respiration had become sufficiently potent to stimulate all the muscles of respiration. The consequence is, that a larger or smaller portion of the lung remains in the state it existed in, in the fœtus. Among the special causes of this imperfect respiration, which, though various, nevertheless preserved the same features in all cases, Jorg enumerates the following:—

1. When the want of oxygen is feebly felt, and consequently only a feeble effort is made in respiring. This may occasionally take place when the delivery has been hurried and easy.

2. When the spinal cord or the brain has been injured by pressure or otherwise, during parturition, and consequently the nervous action of the respiratory organs is suppressed, or is more or less paralyzed.

3. When foreign bodies, such as blood, vaginal mucus, etc., have become introduced into the trachea, or the mouth and nostrils have been closed, or the abdomen and thorax have been compressed.

4. In cases of delivery in the open air, in stables, or other exposed places, where the child is subjected to the influence of sudden cold, and which causes a contraction of the bronchia.

If the first inspirations from any of the above-mentioned causes take place without force and superficially, the air does not pentrate all the cells of the lung, and consequently a portion remains intact, and in the condition in which it existed during intra-uterine life. Sometimes one-half of the lung, or more or less, may remain in this condition.

In making an autopsy of children who have died in consequence of this lesion, those portions of the lung which have remained in the fœtal state for several days after delivery, are

*Ed. Jorg, de morbo pulmonum organico ex respiratione neonatorum imperfecta orto.—Lipsia, 1832.

readily distinguished by their color from those into which air has penetrated, and which are in constant use. In the former, it is dark brown, and has a compact appearance, while in the latter it presents the normal light red, or brick color, and a spongy appearance. Where the two parts border upon each other, the color draws a distinct line between them; the former possesses all the characteristics which it presents in the fœtus, and in structure resembles the liver or spleen; on cutting into it, no crepitation whatever can be perceived, and no air-bubbles arise when the cut portion is pressed under water. If these parts be separated carefully from those into which air has passed, and placed in water, they immediately sink. In infants which have died eight or ten days after birth from this defect, portions of the solid lung may be inflated through the bronchial tubes which lead to them. After having been inflated, the lung will have all the appearances of a normal one. Considerable effort, however, is required to inflate all the cells completely. Occasionally, it happens that empty cells are interspersed with inflated ones over the whole surface of the lung, in which case the latter has a variegated appearance, each part retaining its proper color. In this case, the tissue is more spongy and floats in water, though not upon the surface. Such a lung will only receive its natural color when all the cells are inflated with some force.

It is of the highest importance to know whether any portion of the lung remains in this condition, after being exposed to the current of air which is passing through the healthy portions for several hours or days, and when the child has strength enough and is compelled to make full inspirations. It appears that the ingress of fresh air into the previously opened and enlarged cells gradually compresses the empty ones more and more, and finally obliterates them completely. In the course of ten, twelve, or fifteen days these cells are converted, without any inflammatory process whatever, into a new substance, which has generally been mistaken for hepatized lung. The substance of a respirable lung may certainly become obliterated in consequence of inflammation, but the structure produced by this process differs materially from that which remains of the fœtal lung, the cells of which never have been distended with air. In the latter, we find neither the cells nor the vessels filled with the effused lymph. Its substance is less hard to the touch, and has a peculiar dark-red color. Above all, such lungs do not possess the normal size; and if we, therefore, find cases where the organs of respiration are too small and partially obliterated, we may suspect that imperfect respiration and organic closure of the air-cells, without inflammation, may have produced this defect.

The symptoms which manifest themselves differ somewhat according to the cause which has given rise to the defect. If the infant has been born too quick, it remains (after having commenced to take short and superficial inspirations immediately after being born, and has in no wise shown itself asphyctic,) rather quiet and weak, moves the limbs very little, cries only rarely, and with a weak whining voice; evincing little desire and strength in suckling. During the short and superficial respirations the thorax scarcely moves. In the warm bath the movements of the limbs, and also the voice, become more active, the prostration becomes less, the color of the skin becoming more natural; but the breathing remains the same as before; and after the infant is removed from the bath a short time, all the former symptoms return more or less marked.

If pressure upon the brain or spinal cord (which may result during difficult labor, or from the use of instruments) has been the cause of the atelectasis, the child is generally in a totally asphyctic state for some time, or it may show signs of life; but these are very slight. The breathing may be sighing, but no movement of the extremities takes place. But even if the respiration is active, it is only superficial, and the movement of the thorax imperceptible. It is some time before the infant cries, and then in a weak, trembling tone; the skin remains flaccid, and has a pale or bluish color. If foreign bodies in the trachea, or excessive cold, prevent a normal respiration, we find added to the above symptoms considerable rattling in the air-passages, and even forcible efforts by the child to remove the offending substance. If, during the first efforts of respiration, the air passes only into a small portion of the lung, which frequently happens when a violent impression has been made upon the brain or spinal cord, the respiration may become fuller after the asphyctic state has passed away; but still, it will never have the depth and fulness which are found in healthy infants. In consequence of this, the anterior wall of the thorax does not expand, but retains the flatness which is characteristic of the fœtus.

Infants affected with this organic lesion have a very pale appearance, the color only changing when convulsive paroxysms set in; it then becomes of a bluish purple; the extremities are moved very little; the voice is hoarse and whining; they possess but little strength to suck, and are hardly capable of swallowing the milk which may be given to them. The eyes are closed the greater part of the time, or they may remain open much longer than in healthy infants during the first hours or days after birth. The eyeball is generally drawn inward, and the pupils largely dilated. Such little sufferers rarely enjoy sleep; they are either in a soporose condition, or in a perfectly quiet state, with opened and fixed eyes. Clonic and tonic spasms disturb them in this state of extreme exhaustion. All patients of this class generally die during the first four or five days after birth, either by suffocation or apoplexy. Usually the fatal issue is attributed to mechanical injury of the brain, and the lesion in the lungs is entirely overlooked. When the atelectasis exists only in a small portion of the lung, perhaps one-fourth or less, the diagnosis is more difficult. Exclusive of the paleness, flaccidity, and unnatural coolness and clamminess of the skin, the short and superficial breathing, which constantly accompany this lesion, one might be inclined to consider such infants healthy; and we are the more apt to be deceived, as the characteristic symptoms may not be observed immediately. Such children take the breast regularly, but nevertheless are less nourished than infants that are perfectly healthy. During, as well as after suckling, the skin suddenly assumes a grayish blue, or bluish-red color, which is particularly marked around the mouth. If the voice was nearly natural immediately after birth, it gradually loses its force, and becomes hoarse. Gradually or suddenly the infant loses its desire for suckling, and with this, no matter if it be several days after the infant's birth, the more important symptoms are ushered in, which plainly show the danger, but not the true nature of the disease to the unacquainted; while the body suddenly assumes a bluish color, spasms in the muscles of the face, or of whole groups in the extremities or trunk appear, and threaten the patient with suffocation. These convulsions, which occasionally alternate with those of a tonic character, last a few minutes, disappear entirely, and generally end fatally after the second or third attack; rarely during the first. During these paroxysms the nostrils are widely dilated, the eye is fixed, drawn upward and inward permanently fixed in this position. The eyelids are either entirely closed or widely opened. The voice, during a paroxysm, is hoarse and croup-like; the skin perspires freely, and is cold and livid. With the cessation of the convulsions the warmth returns to the skin; the head and limbs resume their natural position; the patient is able to take the breast for a few moments; but the respiration remains hurried, and the prostration is increased. If by proper treatment, the first paroxysm be alleviated, the following one, which generally appears after twenty-four hours, is less violent; the same with the third, until they cease entirely. The improvement which takes place indicates itself by an unembarrassed respiration, by the disappearance of the hoarseness and bronchial rattling, by easy deglutition and suckling, and by the return of the natural color and warmth of the skin.

*Diagnosis.*—The diagnosis is not very difficult, as the abnormal respiration immediately points to the seat of the disease. The unfailing signs that larger or smaller portions of the lung, without previous inflammation, are compact and inaccessible to the circulation of blood and air, are a short, superficial, anxious, at times scarcely perceivable respiration; a weak, whining voice; little or no desire for the breast; thorax nearly immovable, &c.

The interference with the proper oxidation and prevention of the proper circulation of the blood, in consequence of this organic lesion of the lungs, denotes itself by the bluish, and, during any exertion or crying, dark purple color of the skin and diminished warmth; a weak, slow pulse; and general exhaustion. Convulsions may be either complications or consequences of the above state. The progress of the disease manifests itself by increased difficulty in respiring; short and rapid inspirations; a weak and whispering voice, anxious and steady exertion to enlarge the thorax; inability of deglutition; sympathetic affection of the brain; aggravation of the symptoms of impaired circulation, etc.

The later, however, the physician is called, after the complaint has existed for some time, and the less he can inform himself of the probable cause, the more difficult will be the diagnosis, on account of the different effects of the disease, and the various complications. On applying the stethoscope, the murmur which the air produces in passing through the lungs is only indistinctly heard. Where a larger or smaller lobe of the lung has taken no part in the respiratory process, the sound is cut off at this point completely.

*Treatment*—Few diseases sooner become incurable than, and therefore require such prompt treatment as, the pneumo-atelectasis. If medical aid is called for during the first few hours after the infant's birth, the first indication is to make those cells which are closed respirable. This object may be obtained by general stimulation of the skin, particularly by warm baths, either alone or with the addition of some stimulating substance, such as herba mentha, flor. chamom., etc. These may be previously infused in hot water and then added. During the use of these baths, which should be repeated every two hours until the object is attained, strong friction should be applied over the whole body. The soles of the feet should be rubbed with a flesh-brush until the child makes violent efforts to cry loud. Cold water may also be thrown upon the region of the diaphragm with a syringe, or ether may be sprinkled over the thorax, the infant always being lifted above the surface of the water. The nostrils and fauces may also be irritated with a feather, and enemata of water, with a few drops of ether, applied. Upon taking the infant out of the bath, it should be carefully wrapped up, but in such a manner that no part of the trunk is embarrassed by it. To keep up the irritation which has been excited by the friction and the baths, a small sinapism, two or three inches long and one broad, may be applied across the chest, and allowed to remain until the skin becomes red and irritated. The above treatment will be greatly aided by the appropriate administration of internal remedies. Of these, emetics, or, if these are contra-indicated by an affection of the brain, purgatives will be found of great value. Of the former, the honey of squills, or a powder composed of half a grain of ipecacuanha, with the same amount of sugar and magnesia, may be given until free vomiting takes place. Of the latter, a quarter of a grain of calomel may be given every two hours until free evacuations, with some tenesmus, are produced. The treatment is always aided by the frequent suckling of the infant; the more it is compelled to draw the milk from the breast of the mother, the more influential its own exertions are to a complete cure. If, however, all our attempts to relieve the patient are in vain, on account of adhesions and complete obliteration of the cells of the lung, the chief endeavor should be to alleviate, as much as possible, the condition of the patient. The skin of an infant absorbs oxygen, and consequently, even under such circumstances, a sufficient quantity will be absorbed and carried to the blood to sustain life. Our chief attention should, therefore, be directed to the skin, to keep it in its normal condition. For this end, the infant should always be dressed in loose and thin apparel, and care be taken to embarrass no part of the body. It should be kept in a warm and pure atmosphere, the bowels should be kept in a relaxed condition, and everything liable to cause an acceleration of the respiration carefully avoided, if we would prevent violent suffocatory paroxysms. The infant should be bathed two or three times daily in warm water, to which a small portion of vinegar may be added. Other remedies should be employed, as indications may demand.—*Louisville Review.*

